# A comparative analysis of binary and multi-class classification machine learning algorithms to detect current frailty status using the English longitudinal study of ageing (ELSA)

**DOI:** 10.3389/fragi.2025.1501168

**Published:** 2025-04-22

**Authors:** Charmayne Mary Lee Hughes, Yan Zhang, Ali Pourhossein, Terezia Jurasova

**Affiliations:** Age-Appropriate Human-Machine Systems, Institute of Psychology and Ergonomics, Technische Universität Berlin, Berlin, Germany

**Keywords:** machine learning, frailty, healthcare, elderly, aging, ELSA

## Abstract

**Background:**

Physical frailty is a pressing public health issue that significantly increases the risk of disability, hospitalization, and mortality. Early and accurate detection of frailty is essential for timely intervention, reducing its widespread impact on healthcare systems, social support networks, and economic stability.

**Objective:**

This study aimed to classify frailty status into binary (frail vs. non-frail) and multi-class (frail vs. pre-frail vs. non-frail) categories. The goal was to detect and classify frailty status at a specific point in time. Model development and internal validation were conducted using data from wave 8 of the English Longitudinal Study of Ageing (ELSA), with external validation using wave 6 data to assess model generalizability.

**Methods:**

Nine classification algorithms, including Logistic Regression, Random Forest, K-nearest Neighbor, Gradient Boosting, AdaBoost, XGBoost, LightGBM, CatBoost, and Multi-Layer Perceptron, were evaluated and their performance compared.

**Results:**

CatBoost demonstrated the best overall performance in binary classification, achieving high recall (0.951), balanced accuracy (0.928), and the lowest Brier score (0.049) on the internal validation set, and maintaining strong performance externally with a recall of 0.950, balanced accuracy of 0.913, and F1-score of 0.951. Multi-class classification was more challenging, with Gradient Boosting emerging as the top model, achieving the highest recall (0.666) and precision (0.663) on the external validation set, with a strong F1-score (0.664) and reasonable calibration (Brier Score = 0.223).

**Conclusion:**

Machine learning algorithms show promise for the detection of current frailty status, particularly in binary classification. However, distinguishing between frailty subcategories remains challenging, highlighting the need for improved models and feature selection strategies to enhance multi-class classification accuracy.

## 1 Introduction

With advanced age comes an increasing prevalence of chronic diseases (e.g., diabetes, hypertension, stroke, musculoskeletal disease) and decline in physiological (including physical and cognitive) functions. The concept of physical frailty is of particular interest to the scientific and clinical communities and is characterized by a decline in functioning across multiple physiological systems and accompanied by an elevated vulnerability to stressors (Hoogendijk et al., 2019). Frailty prevalence varies widely based on classification criteria, participant demographics, study settings (e.g., community-dwelling, nursing homes, hospitals), and geographic location, making cross-population comparisons challenging. For instance, a study using data from the Survey of Health, Aging, and Retirement in Europe (SHARE) across 18 countries estimated frailty prevalence at 7.7% (range: 3.0%–15.6%) and pre-frailty at 42.9% (range: 34.0%–52.8%) (Manfredi et al., 2019). Similarly, a recent meta-analysis of community-dwelling older adults reported pooled estimates of 18.1% (95% CI: 13.0%–23.2%) for frailty and 48.9% (95% CI: 43.1%–54.6%) for pre-frailty (Cai et al., 2025). Frail individuals (and to a lesser extent, those who are pre-frail) face an elevated risk for several adverse health outcomes such as comorbidity, disability, dependency, institutionalization, falls, fractures, hospitalization, and mortality (Boyd et al., 2005). Unsurprisingly, frailty is a growing public health concern with significant implications for labor markets, social insurance, pension systems, and healthcare infrastructures (Hooyman and Asuman Kiyak 2013; Makizako et al., 2021).

Frailty is not a progressive condition, but rather a dynamic process in which individuals can transition from a “robust” non-frail state to either a pre-frail or frail state, or conversely transition from a frail or pre-frail state back to a more robust condition (*cf.* Hoogendijk and Dent, 2022). Indeed, there is evidence indicating that frailty is a potentially modifiable dynamic process that can be delayed or reversed with appropriate interventions and health strategies (Fried et al., 2001; Gill et al., 2006; Travers et al., 2019), but this requires accurately detecting the underlying dysregulation of multiple physiological systems associated with frailty before the deterioration of physical functioning has progressed to a level that can be overtly detected.

The use of machine learning (ML) presents a promising solution for enhancing the identification of frailty in older adults, but its effectiveness relies on the availability of sufficient and high-quality data to train accurate and reliable models. A recent systematic review (Leghissa et al., 2023) indicated that more than half of ML-based current frailty status detection studies have utilized electronic health records (EHR) (Gomez-Cabrero et al., 2021; Koo et al., 2022; Le Pogam et al., 2022) or longitudinal aging studies (da Cunha Leme and de Oliveira, 2023) to train the models, although some studies have utilized data from residential care records (Sajeev et al., 2022), or sensor data (Garcia-Moreno et al., 2020, Kim et al., 2020; Razjouyan et al., 2018).

The studies referenced have primarily focused on the predictive performance of binary classification models that distinguish between two levels of frailty, such as non-frail *versus* frail (da Cunha Leme and de Oliveira, 2023; Koo et al., 2022; Le Pogam et al., 2022; Sajeev et al., 2022) or by aggregating two frailty classes together (e.g., grouping frail and pre-frail classes) (Leghissa et al., 2024). ML approaches often simplify multi-class classification problems by reducing them to binary models or by focusing on selected classes. This simplification leverages the computational efficiency and simplicity of algorithms like Support Vector Machines and Logistic Regression and addresses challenges such as class imbalance to enhance performance.

While these reasons certainly have their merit, this approach has notable drawbacks. One significant issue is the loss of information, as collapsing multiple classes into binary categories obscures important distinctions between them. Additionally, binary models may lack sensitivity by failing to capture the full complexity of the data. For example, a model distinguishing only between frail and non-frail individuals might miss critical details within the pre-frail category, leading to less accurate predictions for specific sub-groups. Binary models also often offer reduced interpretability compared to multi-class models. While multi-class models provide detailed insights into the risk or probability of each specific class, binary models may not offer such granularity, making it more challenging to understand and act upon predictions. This can also impact the generalizability of the model, as binary classification models that perform well in one context might not be as effective in other settings that include a broader range of classes. Finally, binary models may oversimplify decision boundaries, resulting in less accurate or meaningful outcomes for complex datasets.

In light of these challenges, this study aimed to evaluate the performance of various ML algorithms, including Logistic Regression, Random Forest, Support Vector Machine, K-Nearest Neighbors, XGBoost, AdaBoost, LightGBM, CatBoost, and Multi-Layer Perceptron, for detecting current frailty status. This assessment was conducted for both binary outcomes (frail vs. non-frail) and multi-class outcomes (frail vs. pre-frail vs. non-frail) using wave 8 of the English Longitudinal Study of Ageing (ELSA) for model development and internal validation, with wave 6 data used for external validation.

## 2 Methods

### 2.1 Study population

This study utilized data from the English Longitudinal Study of Ageing (ELSA), an ongoing prospective cohort survey designed to investigate the health, socioeconomic status, and quality of life of individuals aged 50 years and older residing in English communities as they age. Health Survey for England (HSE) participants are randomly selected to ensure a comprehensive and accurate portrayal of individuals residing in private households across England. Participants were originally recruited from the HSE between 1998 and 2000 (Steptoe et al., 2013), with biannual follow-ups conducted thereafter. To maintain representativeness, refreshment samples (top-ups) were introduced at subsequent waves, ensuring the cohort continued to reflect the aging population of England. The most recent wave (wave 11) commenced in 2023. The protocols for this cohort survey were approved by the South Central–Berkshire Research Ethics Committee (wave 8, approval number: 15/SC/0526, date approved: 23 September 2015) and the NRES Committee South Central - Berkshire (wave 6, approval number: 11/SC/0374, date approved: 28 November 2012). Written informed consent was obtained from all participants at each wave.

### 2.2 Frailty case ascertainment

Frailty status was assessed using Fried’s Frailty Phenotype criteria (Fried et al., 2001), with slight modifications to align with the available data in the ELSA dataset. The five key components - unintentional weight loss, exhaustion, low physical activity, slow walking speed, and weak grip strength - were used to classify frailty. Due to data limitations, information on weight loss in the last 12 months was unavailable in ELSA. Consistent with prior work (Crow et al., 2019; Leghissa et al., 2024), this component was adapted to being underweight, defined as having a body mass index (BMI) of less than 18.5 kg/m_2_ in the current wave. Exhaustion was assessed using two items from the eight-item Center for Epidemiological Studies Depression (CES-D) scale, focusing on feelings of effort and difficulty in initiating activities during the past week. Weakness was determined as grip strength in the lowest 20% of dominant hand values, adjusted for sex and BMI quartiles, or if the respondent had no use of either hand for the grip strength tests or did not complete any of the tests. Slow gait speed was defined as being in the lowest 20% of gait speed values (adjusted for sex and median height), or if the respondent was not able to complete any of the walking tests. Low physical activity was determined based on self-reported frequency, with the lowest category (“hardly ever or never”) indicating low physical activity. It is important to note that in ELSA, key components such as grip strength and BMI (and the variables used to calculate BMI, i.e., height, weight) are collected only in even-numbered waves (i.e., waves 2, 4, 6, 8, and 10). Each criterion was assigned a numerical score of 1 if met and 0 if not, and the frailty score was derived by summing the scores of the five criteria (range 0–5). Participants were classified as non-frail (0 criteria), pre-frail (1–2 criteria), or frail (3–5 criteria).

### 2.3 Feature engineering

**FIGURE 1:**
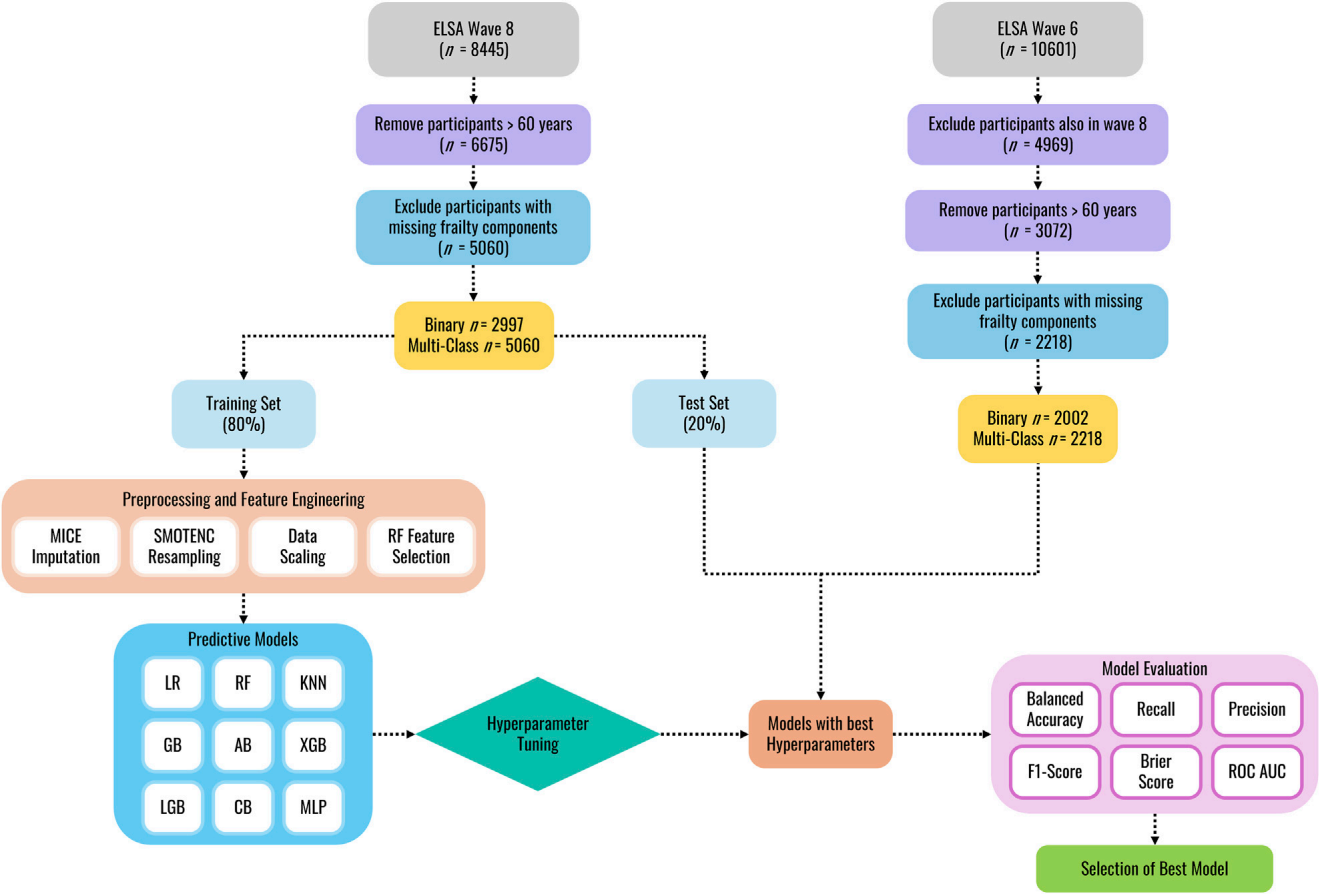
Machine learning classification workflow.

The data processing workflow is shown in Figure 1. A multi-step procedure was used to select key variables associated with frailty from the ELSA data set. First, the selection of potential explanatory variables was narrowed down by removing participants younger than 60 years of age (as only individuals 60 years and above performed the gait task), variables related to the respondent’s spouse, and then removing variables with missingness greater than 30%. Second, since some variables in the ELSA dataset change across waves, only variables/features that were present in both wave 6 and wave 8 were included in the feature selection process, ensuring consistency in the data used for training and validation. Third, multicollinearity was addressed in the wave 8 dataset by first removing highly correlated variables (*r* > 0.80), then removing variables with a variance inflation factor (VIF) above 5.

Following initial data cleaning, the wave 8 dataset was randomly split into training and internal validation sets (80:20 ratio) using stratified random splitting to preserve class distributions and prevent data leakage. Missing data, identified as non-random by Little’s MCAR test (p < 0.01; Li, 2013), were addressed via Multivariate Imputation by Chained Equations (MICE). This method generates imputed values by drawing from estimated conditional distributions of the variable with missing data given all other variables in the dataset (Van Buuren and Oudshoorn, 1999). The MICE imputation model was fitted exclusively on the wave 8 training data. The trained imputer was then used to impute missing values in both the internal test set and the external wave 6 validation data, ensuring consistency in the imputation process without data leakage. To address class imbalance in the training data, the Synthetic Minority Over-Sampling Technique for Nominal and Continuous features (SMOTENC, Chawla et al., 2002) was applied solely to the imputed training data, generating synthetic samples of the minority class while preserving categorical data structures. Following resampling, StandardScaler was fitted on the training data (after resampling) to standardize input features (mean = 0, standard deviation = 1). The learned mean and standard deviation parameters were then applied to standardize both the unmodified internal test and external validation sets. Feature selection was performed after scaling using a Random Forest-based embedded method (Breiman, 2001) to identify predictive features from the standardized training data. Feature importance scores were computed, and the smallest subset of features contributing to 95% of cumulative importance was retained to ensure selection reflected scaled feature contributions.

Model performance was evaluated using tenfold cross-validation on the resampled training set. In each iteration, the training data was split into 90% for model fitting and 10% for validation, ensuring that preprocessing steps were independently refit on the 90% subset to prevent leakage. For external validation, participants from wave 6 (who were not included in the training or internal validation) were used to assess generalizability. These participants’ data underwent preprocessing using parameters learned from the training set, preserving real-world applicability while maintaining strict separation from the model development process.

### 2.4 Statistical analysis

To analyze the differences between groups, traditional statistics were employed separately for Wave 8 (training and testing data) and Wave 6 (validation data) to assess the distribution and group characteristics before applying ML models. The normality of the data for each wave was first evaluated using the Shapiro-Wilk test. Given that the data did not follow a normal distribution (*p* < 0.05), separate non-parametric Kruskal–Wallis H tests were performed to evaluate whether statistically significant differences between the medians of the frailty statuses exist. The Kruskal–Wallis test does not assume normal distribution and is suitable for comparing more than two independent groups. For the multi-class context, Dunn’s test for pairwise comparisons was conducted to identify which specific groups differed from each other following a significant Kruskal–Wallis test result. Dunn’s test is a non-parametric *post hoc* analysis suitable for multiple comparisons and provides adjusted *p*-values to control for the Type I error rate. The results of Dunn’s tests provided detailed insights into pairwise group differences, offering a robust follow-up analysis to the initial Kruskal–Wallis test. A *p*-value of less than 0.05 was considered indicative of a statistically significant difference.

### 2.5 Machine learning models

After feature selection, 9 ML models were developed to detect current frailty status in the ELSA dataset: Logistic Regression, Random Forest, K-Nearest Neighbors, Gradient Boosting, AdaBoost, XGBoost, LightGBM, CatBoost, and Multi-layer Perceptron. The utilization of these models allowed for the exploration of a wide range of techniques for frailty classification and offer valuable insights into this critical health issue.

• *Logistic Regression:* A linear model for binary classification, which predicts the probability of a class by modeling the log-odds of the outcome using a logistic function. For multi-class classification, it can be extended through approaches like one-vs-rest or softmax regression (multinomial logistic regression). It assumes linearity between the features and log-odds and is particularly useful when interpretability is key.
• *Random Forest:* An ensemble learning method for classification that builds a collection of decision trees using randomly selected subsets of data and features. Each tree makes a class prediction, and the final prediction is made by majority voting across all trees. Random Forest reduces variance and overfitting while being robust to noise, and it can handle both binary and multi-class classification tasks.
• *K-Nearest Neighbor:* A non-parametric classification algorithm that assigns a class label based on the majority class of the *k*-nearest neighbors in the feature space. The classification decision is based on a distance metric, such as Euclidean or Manhattan distance. K-Nearest Neighbors is sensitive to the choice of *k* and the scale of the features but performs well when the decision boundary is non-linear.
• *Gradient Boosting*: A sequential ensemble method that iteratively combines weak learners (shallow decision trees) to build a strong predictive model. Each iteration fits a new learner to the residual errors of the current ensemble, minimizing a loss function through gradient descent. This approach reduces bias and prevents overfitting through regularization techniques, making it suitable for both regression and classification tasks, especially in datasets with complex relationships.
• *XGBoost:* A gradient boosting algorithm designed for classification tasks, building decision trees sequentially, where each tree corrects the errors of the previous one. For classification, it outputs a probability for each class and then selects the class with the highest probability. XGBoost excels in handling complex non-linear relationships and provides strong regularization (L1 and L2) to prevent overfitting, making it highly effective for binary and multi-class classification.
• *AdaBoost:* A boosting algorithm that combines weak learners (usually shallow decision trees) by focusing more on misclassified instances in each subsequent iteration. It assigns higher weights to misclassified points, iteratively adjusting the learners to reduce the overall error. The final model is a weighted combination of all weak learners, effectively improving model performance.
• *LightGBM:* A highly efficient gradient boosting algorithm that excels in classification tasks by constructing decision trees using a histogram-based method. LightGBM uses a leaf-wise growth strategy, which reduces error faster but can lead to overfitting if not controlled with regularization. It is optimized for speed and scalability, making it suitable for both binary and multi-class classification problems.
• *CatBoost:* A gradient boosting algorithm specifically designed for handling categorical features effectively. In classification tasks, CatBoost handles multi-class and binary classification by creating a series of decision trees that correct the errors of the previous models. It uses techniques like ordered boosting and random permutations to avoid target leakage, improving performance and reducing overfitting in classification tasks with categorical variables.
• *Multi-Layer Perceptron:* A feedforward artificial neural network used for both binary and multi-class classification. In an MLP, multiple hidden layers with non-linear activation functions (e.g., ReLU or tanh) enable the network to learn complex patterns in data. The final output layer applies a softmax function for multi-class classification or a sigmoid function for binary classification. MLPs are highly flexible but require extensive tuning to avoid overfitting, especially in classification tasks.

### 2.6 Hyperparameter tuning

To optimize the performance of the ML models, hyperparameter tuning was conducted using GridSearchCV. GridSearchCV is an exhaustive search method that evaluates a predefined set of hyperparameters to identify the optimal combination that maximizes model performance. For each model, we specified a range of hyperparameters based on common practice and prior research (Supplementary Table S7). During the tuning process, GridSearchCV iteratively trained each model on a training set using each possible combination of hyperparameters. A tenfold cross-validation technique was employed to evaluate the performance of each configuration. The performance of each model configuration was assessed using the same metrics outlined in Section 2.8. The hyperparameter set yielding the highest cross-validation score was selected as the optimal configuration for each model.

### 2.7 External validation

External validation is a critical phase in model development, as it evaluates the model’s generalizability and estimates its performance on an independent population. In this study, models were developed and internally validated using wave 8 of the ELSA dataset, while external validation was conducted on wave 6, collected 4 years earlier. To ensure the independence of the external validation set, participants who appeared in both waves were excluded. Additionally, variables were restricted to those available in both waves, ensuring consistency across datasets. External validation was performed on the hyperparameter-tuned models developed from wave 8 data.

### 2.8 Model evaluation

The primary performance metrics of interest included balanced accuracy, recall/sensitivity, precision, the F1 score, and the Brier score (Hossin and Sulaiman, 2015). Balanced accuracy is defined as the mean of sensitivity and specificity, providing a robust performance metric for imbalanced datasets by equally weighting classification performance across all classes. Recall relates to the proportion of actual positive cases that the classifier correctly identifies as data and reflects the number of positive predictions that the classifier correctly identifies. Precision relates to the percentage of predicted positives that are actually correct and reflects the classifier’s ability to minimize false positive predictions. The F1 score is the harmonic mean of precision and recall, with higher F1 scores indicating a better prediction performance. Lastly, the Brier score measures the mean squared difference between predicted probabilities and the actual outcomes. It is used to assess the accuracy of probabilistic predictions, with lower Brier scores indicating better-calibrated predictions.

In addition, each model was quantitatively evaluated using the Area Under the Receiver Operating Characteristic Curve (ROC-AUC) score. The ROC curve demonstrates a model’s ability to discriminate between a specific class and all others in multi-class settings, and between positive and negative classes in binary classification, across different decision thresholds. A high-performing model will have an ROC curve that covers a larger area under the curve (AUC), ideally approaching the top-left corner (0, 1), indicating near-perfect discrimination. The evaluation performance of the AUC value was defined by Hosmer et al. (2013): AUC ≥0.9, outstanding discrimination; 0.8 ≤ AUC <0.9, good discrimination; 0.7 ≤ AUC <0.8, acceptable/fair discrimination; 0.6 ≤ AUC <0.7, poor discrimination; and AUC <0.6, no discrimination.

### 2.9 Implementation details

The models were executed on a Windows 11 Pro machine containing an AMD Ryzen 9 7940HS processor with 64 GB of RAM. All codes were written in Python version 3.11.5. Models were implemented using scikit-learn (Pedregosa et al., 2011), XGBoost 2.1.0 (Chen and Guestrin, 2016), and/or CatBoost 1.2.7 (Yandex, 2024). Statistical analyses were computed using SciPy (Virtanen et al., 2020) and scikit_posthoc (Terpilowski, 2019) packages.

## 3 Results

### 3.1 Baseline differences between included and excluded participants

To assess potential baseline differences between included and excluded participants, chi-square tests were conducted for categorical variables (gender, race, education level, marital status, and place of birth), and t-tests were used for continuous variables (i.e., age). Full details are provided in the Supplementary Material (Tables 1, 2).

**TABLE 1:** Comparison of model performance with two classes (non-frail, frail).

	Balanced accuracy	Recall	Precision	F1-score	Brier score	ROC AUC
Internal validation						
Logistic Regression	0.930	0.940	0.943	0.941	0.060	0.981
Random Forest	0.921	0.950	0.949	0.949	0.050	0.977
K-Nearest Neighbor	0.872	0.920	0.918	0.918	0.080	0.920
Gradient Boosting	0.923	0.947	0.947	0.947	0.053	0.980
AdaBoost	0.932	0.949	0.950	0.949	0.051	0.980
XGBoost	0.926	0.948	0.948	0.948	0.052	0.980
LightGBM	0.915	0.945	0.944	0.944	0.055	0.981
CatBoost	0.928	0.951	0.951	0.951	0.049	0.980
Multi-layer Perceptron	0.910	0.934	0.935	0.934	0.066	0.971
External Validation						
Logistic Regression	0.932	0.942	0.950	0.944	0.058	0.971
Random Forest	0.897	0.947	0.947	0.946	0.053	0.960
K-Nearest Neighbor	0.947	0.947	0.946	0.946	0.053	0.921
Gradient Boosting	0.884	0.940	0.941	0.941	0.060	0.960
AdaBoost	0.915	0.945	0.949	0.946	0.055	0.965
XGBoost	0.904	0.950	0.951	0.950	0.050	0.962
LightGBM	0.914	0.952	0.953	0.952	0.048	0.966
CatBoost	0.913	0.950	0.952	0.951	0.050	0.970
Multi-layer Perceptron	0.916	0.947	0.950	0.948	0.053	0.966

**TABLE 2:** Comparison of model performance with three classes (non-frail, pre-frail, frail).

	Balanced accuracy	Recall	Precision	F1-score	Brier score	ROC AUC
Internal validation						
Logistic Regression	0.669	0.642	0.661	0.664	0.239	0.841
Random Forest	0.684	0.688	0.695	0.691	0.208	0.853
K-Nearest Neighbor	0.621	0.590	0.598	0.580	0.273	0.780
Gradient Boosting	0.654	0.670	0.674	0.671	0.220	0.841
AdaBoost	0.680	0.668	0.675	0.670	0.221	0.799
XGBoost	0.660	0.664	0.670	0.666	0.224	0.844
LightGBM	0.661	0.667	0.673	0.670	0.221	0.847
CatBoost	0.673	0.668	0.674	0.670	0.221	0.847
Multi-layer Perceptron	0.669	0.642	0.661	0.645	0.239	0.838
External Validation						
Logistic Regression	0.641	0.637	0.648	0.637	0.242	0.820
Random Forest	0.613	0.656	0.656	0.656	0.229	0.823
K-Nearest Neighbor	0.575	0.552	0.551	0.538	0.298	0.747
Gradient Boosting	0.619	0.666	0.663	0.664	0.223	0.812
AdaBoost	0.616	0.630	0.638	0.633	0.246	0.779
XGBoost	0.613	0.663	0.660	0.661	0.225	0.817
LightGBM	0.612	0.654	0.650	0.652	0.231	0.815
CatBoost	0.591	0.650	0.649	0.649	0.233	0.804
Multi-layer Perceptron	0.647	0.632	0.645	0.632	0.245	0.826

In wave 8, included participants had a mean age of 71.5 years *versus* 70.9 years for excluded participants (t = −2.729, *p* = 0.006), but the effect size was negligible (Cohen’s d = −0.076). Males comprised 44.17% of included participants and 46.93% of excluded participants (χ^2^ = 3.67, *p* = 0.055, Cramér’s V = 0.023). A significant difference in racial identity was observed (χ^2^ = 8.64, *p* = 0.003, Cramér’s V = 0.036), though the effect size was small, with similar proportions of participants identifying as white (included: 97.43%, excluded: 95.98%). Education levels also differed significantly (χ^2^ = 25.00, *p* < 0.001, Cramér’s V = 0.061), but the small effect size suggests only modest differences in proportions. Marital status varied significantly (χ^2^ = 12.07, *p* = 0.007), but the small effect size indicates limited practical significance (Cramér’s V = 0.048, e.g., 67.41% vs. 71.21% married/partnered). UK-born participants were more common among included (92.21%) than excluded (89.04%) individuals (χ^2^ = 15.36, *p* < 0.0001), but the small effect size (Cramér’s V = 0.048) suggests minimal practical impact.

In wave 6, excluded participants were significantly older (included: 71.8 years, excluded: 73.49 years, t = −5.622, *p* < 0.001), though the small effect size (Cohen’s d = −0.190) suggests only a modest difference. Gender distribution was similar (47.99% vs. 48.90% male, χ^2^ = 0.259, *p* = 0.611, Cramér’s V = 0.009), as was racial composition (97.20% vs. 96.25% white, χ^2^ = 2.184, *p* = 0.139, Cramér’s V = 0.025). Education levels differed significantly (χ^2^ = 17.632, *p* < 0.001, Cramér’s V = 0.074), though the small effect size suggests only modest differences (e.g., more excluded participants had below upper secondary education: 47.10% vs. 39.76%). Marital status also differed significantly (χ^2^ = 17.582, *p* = 0.001, Cramér’s V = 0.071), but the small effect size suggests limited practical impact (e.g., 19.98% vs. 24.23% widowed). The proportion of UK-born individuals was similar (91.54% vs. 90.52%, χ^2^ = 0.995, *p* = 0.319, Cramér’s V = 0.017).

### 3.2 Dataset characteristics and class distribution for multi-class and binary classifications

A full description of the candidate predictors for both classification approaches is provided in Supplementary Tables S3, S4. For binary classification (frail vs. non-frail), a set of 34 variables was used. These included demographic variables such as age (rwagey) and education level (raeducl). Health-related variables included self-reported general health status (rwshlt), changes in self-reported health (rwshltc), the number of falls in the past 2 years (rwfallnum), difficulties with activities of daily living (rwadlwaa), change in activities of daily living score from the previous to the current interview (rwadlc), gross motor index score (rwgrossa), change in gross motor index score from the previous to the current interview (rwgrossc), the sum of difficulties with instrumental activities of daily living (rwiadlza), difficulties in mobility (rwlowermoba, rwuppermoba), arthritis presence (rwarthre), self-rated vision (rwsight, rwdsight, rwnsight), usual pain level (rwpainlv), and frequency of light physical activity (rwltactx_e). Cognitive function was represented by the total word recall score (rwtr20). Income-related variables such as disability pension income (rwissdi) and public old-age pension (rwisret). Retirement expectations included the respondent’s retirement age (rwretage), the probability of living to older ages (rwliv10), the probability of receiving an inheritance in the next 10 years (rwinher), the probability of moving to a nursing home in the next 5 years (rwpnhm5y). Family structure was captured through membership in organizations or clubs (rwsocyr), assistance and caregiving was captured by whether the respondent provided informal care to grandchildren in the last week (rwgkware1w), and stress was captured by lack of child, other family members, and friends support (rwksupport6, rwosupport6, rwfsupport6). Psychosocial factors were assessed with scales such as life satisfaction (rwlsatsc3), societal position (rwcantril), quality of life (rwcasp19), and depression (rwcesd).

For multi-class classification (frail, pre-frail, non-frail), 57 variables were used. These included demographic variables such as age (rwagey), education level (raeducl), and reported religion (rarelig_e). Health-related variables included the number of falls in the past 2 years (rwfallnum), self-reported health status (rwshlt), changes in self-reported health status from the previous to the current interview (rwshltc), limitations in daily activities (rwadlwaa), changes in self-reported health status from the previous to the current interview (rwadlc), difficulties with instrumented activities of daily living (rwiadlza), gross motor index score (rwgrossa), change in gross motor index score from the previous to the current interview (rwgrossc), difficulties in mobility (rwlowermoba, rwuppermoba), arthritis presence (rwarthre), sensory impairments (rwsight, rwdsight, rwnsight, rwcataracte, rwhearing), urinary incontinence (rwurinai), ever having psychological problems, asthma, high cholesterol, or high blood pressure (rwpsyche, rwasthmae, rwhchole, rwhibpe), sleep issues (rwwakent_e, rwwakeup_e), usual level of pain (rwpainlv), frequency of light physical activity (rwltactx_e), and smoking status (rwsmokev, rwsmokef). Cognitive function was represented by the word recall score (rwtr20) and orientation score (rworient), while income-related variables included employment earnings (rwitern), disability income (rwissdi), and public pension income (rwisret). Employment history variables included labor force status (rwlbrf_e) and whether the respondent is currently working for pay (rwwork). Retirement-related variables such as expected retirement age (rwretage), probability of nursing home place within 5 years (rwpnhm5y), main reason for retiring (rwrets), early retirement status (rwearlyret), and probability of living to an older age (rwliv10) were also included. The number of occupational pensions (rwpeninm) was included from the pension module. Family structure was represented by weekly contact with children and friends (rwkcnt, rwcntpm, rwfcnt, rwfcntpm), and participation in social clubs (rwsocyr). Life insurance coverage (rwlifein) was included from the end-of-life module. Caregiving was represented by the number of grandchildren cared for in the past week (r8gkcare1w). Stress-related variables included family and friend support scores (rwksupport6, rwosupport6, rwfsupport6). Lastly, psychosocial factors included life satisfaction (rwlsatsc3), subjective socioeconomic position (rwcantril), quality of life (rwcasp19), and depression scores (rwcesd).

The characteristics of the datasets and associated statistical significance can be found in the Supplementary Tables S5, S6. There were 34 input features in the binary classification task. In wave 8, non-frail individuals accounted for 85.35% of total cases, and frail individuals accounted for 14.65% of total cases (total *n* = 2997). In wave 6, non-frail individuals accounted for 77.42% of total cases, and frail individuals accounted for 22.58% of total cases (total *n* = 2002). The multi-class classification case consisted of 57 input features. In wave 8, there were 50.55% non-frail individuals, 40.77% pre-frail individuals, and 8.68% frail individuals (total *n* = 5060). In wave 6, 43.73% were considered non-frail, 41.75% pre-frail, and 14.52% frail (total *n* = 2218).

### 3.3 Classification results

Tables 1, 2 show the behavior of the classifiers across multiple performance metrics for the internal and external validation sets for the binary and multi-classification tasks.

#### 3.3.1 Binary classification

**FIGURE 2:**
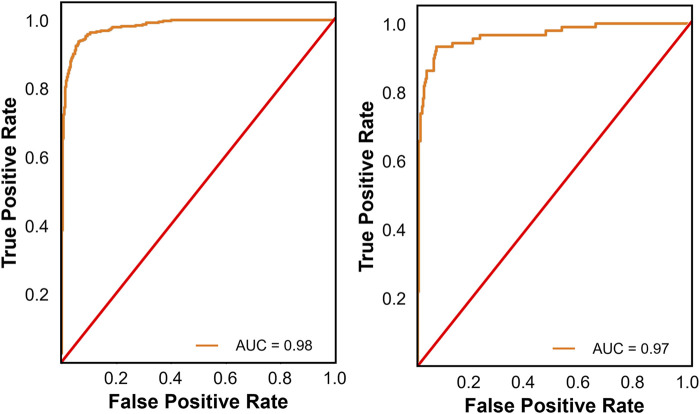
Receiver operating characteristic (ROC) curves for the best performing binary classification model on the internal (left panel) and external validation sets (right panel). The ROC curve illustrates the trade-off between true positive and false positive rates, providing insight into the models’ discrimination ability across different thresholds.

For binary classification (frail vs. non-frail), all nine models demonstrated strong classification performance, with ROC-AUC scores above 0.920 across both validation sets. Among the nine binary classification models evaluated, CatBoost demonstrated the most robust overall performance, achieving high recall (0.951), balanced accuracy (0.928), and ROC-AUC values (0.980, Figure 2, left) on the internal validation set. Externally, it maintained strong classification capability with a recall of 0.950, balanced accuracy of 0.913, an F1-score of 0.951, and an ROC-AUC of 0.970 (Figure 2, right). LightGBM and XGBoost also performed well, with LightGBM achieving the highest precision (0.953) and F1-score (0.952) in external validation. While K-Nearest Neighbor had the highest balanced accuracy externally (0.947), its overall discrimination was weaker compared to other ensemble models. Given its superior external validation performance in terms of recall, calibration, and discrimination, CatBoost emerged as the more robust model for multi-class classification. This performance was attained with a fine-tuned configuration of 200 iterations, a learning rate of 0.1, a maximum tree depth of 6, and an L2 regularization value of 1.

Analysis of the confusion matrix for CatBoost reveals its strengths and weaknesses. In the internal validation set, CatBoost model correctly classified 77 individuals (88.5%) as frail, but misclassified 10 (11.5%) as non-frail. The model demonstrated strong performance in identifying non-frail individuals with 498 (97.1%) correctly classified cases and 15 (2.9%) misclassified as frail. In the external validation set, the model correctly predicted 390 frail cases (86.3%) but misclassified 62 (13.7%) as non-frail. CatBoost performed well in identifying non-frail individuals, achieving high accuracy (1496 correct predictions, 96.5%) with only a small number of misclassifications (54 instances, 3.5%).

#### 3.3.2 Multi-class classification

**FIGURE 3:**
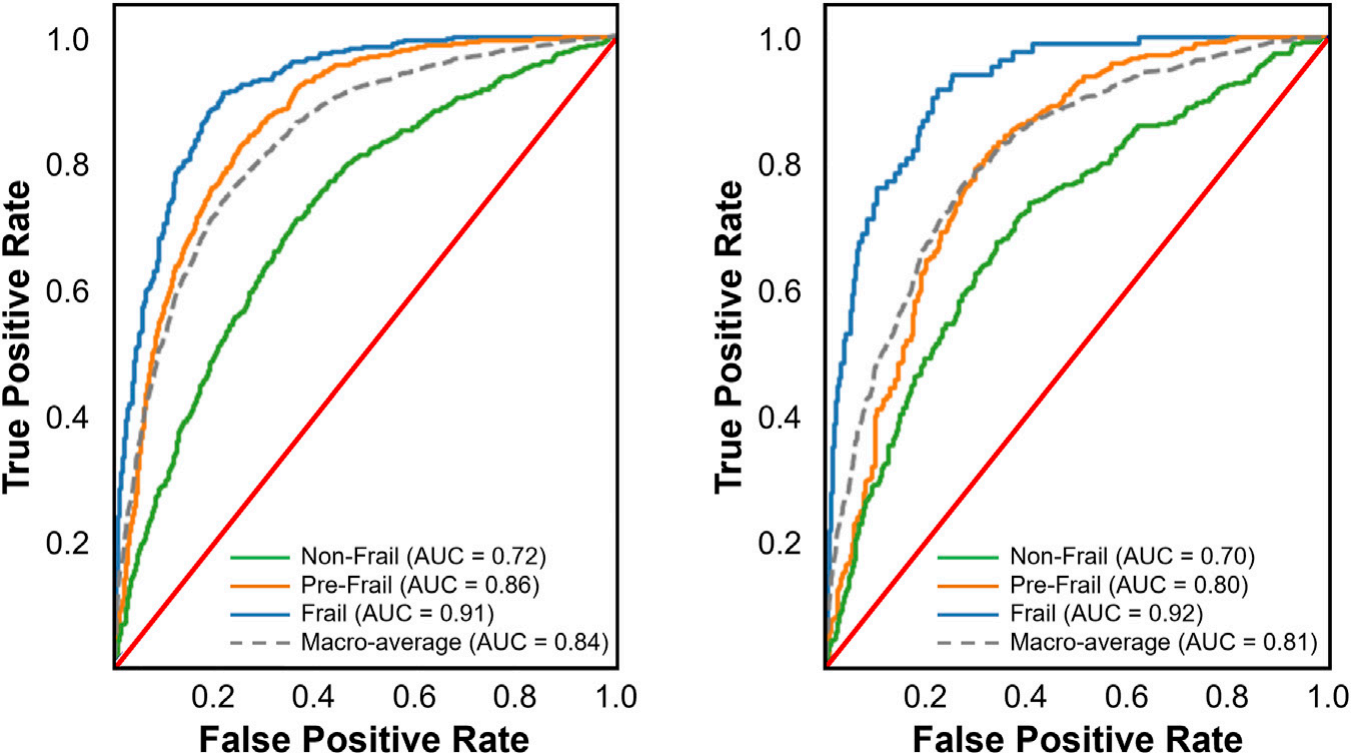
Receiver operating characteristic (ROC) curves for the best performing multi-class classification model on the internal (left panel) and external validation sets (right panel). The multi-class ROC curves include one-vs-all curves for each class along with the macro-average. These curves illustrate the trade-off between true positive and false positive rates, providing insight into the models’ discrimination ability across different thresholds.

Among the nine multi-class classification models evaluated, Random Forest achieved the best performance on the internal validation set, with the highest balanced accuracy (0.684), recall (0.688), F1-score (0.691), and the lowest Brier score (0.208), outperforming models like Logistic Regression, XGBoost, and Gradient Boosting. Gradient Boosting also performed well, with a recall of 0.670, balanced accuracy of 0.654, F1-score of 0.671, and an ROC-AUC of 0.841 (Figure 3, left), showing strong classification capability and better calibration than XGBoost and Logistic Regression. On the external validation set, Random Forest’s performance dropped significantly, with a decrease in balanced accuracy (0.071), recall (0.032), and F1-score (0.035). In contrast, Gradient Boosting achieved the highest recall (0.666) and precision (0.663) on the external validation set, surpassing Random Forest, XGBoost, and LightGBM. While its balanced accuracy (0.619) was lower than some models, its F1-score (0.664) remained strong, and its Brier Score (0.223) and ROC-AUC value (0.812, Figure 3, right) indicated reasonable calibration.

Seven of the nine models demonstrated good discrimination, while KNN and AdaBoost showed only acceptable performance. Random Forest exhibited the strongest and most consistent discrimination (internal = 0.853, external = 0.823), followed by LightGBM (0.847, 0.815) and XGBoost (0.844, 0.817). Gradient Boosting (0.841, 0.812) and Logistic Regression (0.841, 0.820) performed comparably, while CatBoost (0.847, 0.804) showed a notable decline externally. KNN (0.780, 0.747) had the lowest discrimination, and AdaBoost (0.799, 0.779) was the weakest ensemble model. Multi-layer Perceptron performed well internally (0.838) but declined externally (0.826).

Considering both internal and external validation results, Gradient Boosting emerged as the most reliable model, as it maintained strong recall across both datasets. While Random Forest performed well on internal validation, its decline on external data suggests potential overfitting. Thus, Gradient Boosting is the preferred model for frailty classification due to its more stable generalization and ability to identify frail cases consistently. Gradient Boosting’s performance was attained with a fine-tuned configuration of 200 trees, a learning rate of 0.2, a maximum depth of 7, and 0.9 subsamples.

An analysis of the confusion matrix for Gradient Boosting reveals its strengths and weaknesses. For the internal validation set, the model correctly classified 50 individuals (60.2%) as frail, but misclassified 32 (38.6%) as non-frail, and 1 case (1.2%) as pre-frail. The model performed well in identifying pre-frail individuals (381 correct predictions, 72.0%), but with some misclassifications as frail (6 instances, 1.1%) and non-frail (142 instances, 26.8%). Prediction of the non-frail class was also reasonable, with 256 correct predictions (64.0%), 89 instances (22.3%) misclassified as pre-frail, and 55 (13.8%) misclassified as frail. In the external validation set, the Gradient Boosting model correctly predicted 171 (53.0%) frail cases, but misclassified 4 (1.2%) cases as pre-frail, and 147 (45.8%) cases as non-frail. Gradient Boosting excelled at identifying pre-frail individuals, achieving high accuracy (748 correct predictions, 77.1%) but misclassified some as non-frail (218 instances, 22.5%) and frail (4 instances, 0.4%). The model also performs reasonably well for non-frail individuals, correctly predicting 514 cases (55.5%), but with some misclassifications as pre-frail (319 instances, 34.5%) and frail (93 instances, 10.0%).

## 4 Discussion

Physical frailty, with its profound implications for increased disability, hospitalization, and mortality risks, underscores the urgent need for accurate early detection to facilitate timely interventions and mitigate its extensive impacts on healthcare systems and social support networks. To address this need, this study evaluated the performance of various ML algorithms (i.e., Logistic Regression, Random Forest, K-Nearest Neighbors, Gradient Boosting, AdaBoost, XGBoost, LightGBM, CatBoost, and Multi-Layer Perceptron) in detecting current frailty status. Using data from wave 8 of ELSA for model development and internal validation, and wave 6 for external validation, we assessed these algorithms for both binary (frail vs. non-frail) and multi-class classification tasks (frail vs. pre-frail vs. non-frail), aiming to gain a deeper understanding of their effectiveness in accurately detecting frailty status.

The comparison between binary and multi-class classification revealed substantial performance differences. CatBoost excelled in binary classification, achieving strong performance and consistent performance across both internal (balanced accuracy of 92.8%, ROC-AUC of 0.980) and external validation sets (balanced accuracy of 91.3%, ROC-AUC of 0.970). Conversely, multi-class classification presented greater challenges, with the top-performing model, Gradient Boosting, achieving an external validation set balanced accuracy of only 61.9% and a macro-average ROC-AUC of 0.812. This disparity highlights the inherent complexity of distinguishing between multiple classes simultaneously. It is not surprising that different algorithms excelled in binary *versus* multi-class classification tasks, as the decreased performance in the multi-class classification highlights the added complexity of simultaneously distinguishing between multiple categories. The higher performance in binary classification is largely due to the simpler decision boundaries, as models only need to distinguish between frail and non-frail categories. This allows algorithms to focus on a more straightforward optimization problem, leading to higher precision, recall, and calibration. In contrast, multi-class classification requires differentiating among three categories (frail, pre-frail, and non-frail), introducing overlapping class boundaries, a higher likelihood of misclassification, and increased difficulty in optimizing decision thresholds across all classes. Additionally, class imbalance can exacerbate this challenge, as certain frailty categories may be underrepresented, making it harder for models to learn generalizable patterns.

Given these challenges, it is not surprising that different algorithms excelled in binary *versus* multi-class classification tasks. CatBoost emerged as the most robust model, demonstrating strong performance across both internal and external validation sets. Its efficiency in handling categorical data and its use of ordered boosting to reduce data leakage and enhance generalization contributed to its success. Additionally, CatBoost’s regularization techniques and fine-tuned hyperparameters enabled it to model complex decision boundaries while maintaining high calibration and discrimination. These factors made it particularly well-suited for binary classification tasks, where it consistently outperformed other models in recall, balanced accuracy, and ROC-AUC scores.

In the context of multi-class classification, Gradient Boosting emerged as the top model for frailty classification, surpassing Random Forest despite the latter’s strong performance on the internal validation set. While Random Forest demonstrated high accuracy, recall, and F1-score internally, its performance significantly dropped on the external validation set, suggesting potential overfitting to the training data. In contrast, Gradient Boosting’s performance remained more stable across both internal and external sets, indicating better generalization. This robustness is due to Gradient Boosting’s ability to focus on hard-to-classify instances through gradient descent-based optimization, which is particularly important in multi-class tasks where distinguishing between the frail, pre-frail, and non-frail categories requires nuanced decision boundaries. Additionally, the ensemble approach of Gradient Boosting, combining multiple weak learners, allows it to effectively handle the class imbalance and adapt to the complexities of frailty classification. These factors contributed to its superior ability to consistently identify frail individuals, making it the best choice for multi-class frailty classification compared to Random Forest.

To build on the previous discussion of model performance, it is important to recognize the broader context in which these models were applied. Our key strength is the use of external validation, which mitigates overfitting and enhances the generalizability of our models. Additionally, we systematically compared multiple ML models and their performance across both binary and multi-class classification tasks. While our findings offer valuable insights into frailty detection, there are certain limitations inherent in the dataset and the study design that should be acknowledged. One such limitation is that our study focused on detecting current frailty status rather than the prospective prediction of frailty, which could have broader implications for preventive interventions. This approach was necessitated by limitations inherent in the structure of the ELSA dataset. Firstly, the data are collected in waves that are spaced 2 years apart, with key frailty measures like grip strength and BMI captured only in even-numbered waves (i.e., waves 2, 4, 6, 8, and 10). As a result, grip strength and other critical components of frailty are only measured every 4 years, limiting the frequency at which frailty can be assessed and thus making it challenging to track more frequent changes in frailty status. Moreover, the dataset does not include data on weight loss in the past 12 months, which is a core component of frailty as defined by Fried’s Frailty Phenotype. While modifications were made to address these issues, they may not fully reflect dynamic changes in frailty over time, which complicates the task of prospective prediction. Therefore, while the ELSA dataset provides valuable insights into the current state of frailty, it does not offer the continuous, high-resolution data necessary to predict future frailty status with the same accuracy.

To improve frailty classification and potentially overcome these challenges for prospective prediction, leveraging sensor data (Apsega et al., 2020; Akbari et al., 2021; Razjouyan et al., 2018), or digital biomarkers (Gomez-Cabrero et al., 2021; Sargent et al., 2024), alongside the health, social, wellbeing and economic data already collected, offers several promising avenues. Prior research has demonstrated that sensor-based metrics, such as gait speed, balance, and physical activity levels, can provide critical insights into frailty status (Apsega et al., 2020; Razjouyan et al., 2018). Similarly, analyzing time-series data and employing signal processing techniques, such as Fast Fourier Transform or wavelet decomposition, can reveal temporal patterns and extract meaningful features from raw sensor data, thus enhancing model accuracy. Incorporating data from multiple sensor modalities—such as accelerometers, gyroscopes, and heart rate monitors—into ML models can provide a more holistic understanding of an individual’s condition. Additionally, genomics data could reveal biomarkers associated with aging, inflammation, and muscle degeneration, which may signal early frailty before physical symptoms become evident (Gomez-Cabrero et al., 2021; Sargent et al., 2024). By combining biological, physiological, and sensor data, ML models could uncover subtle changes that precede overt behavioral symptoms, allowing for more accurate differentiation between frail, pre-frail, and non-frail individuals. Furthermore, integrating sociodemographic data—such as age, gender, socioeconomic status, and lifestyle factors—collected by the ELSA study could further refine model performance. These factors are closely linked to frailty risk and progression, and their inclusion could enable more precise stratification of participants and facilitate earlier interventions.

## 5 Conclusion

In conclusion, this study highlights the potential of ML algorithms to enhance early detection of physical frailty, a condition associated with increased risks of disability, hospitalization, and mortality. The performance gap between binary and multi-class classification underscores the added complexity of distinguishing multiple frailty states, which involve more intricate decision boundaries and higher misclassification rates. While current models perform well in binary classification, future research should explore multi-modal approaches that incorporate sensor, genomic, and sociodemographic data to enhance predictive accuracy. Such advancements could improve frailty detection and enable earlier, more targeted interventions to mitigate its adverse outcomes.

## Data Availability

Publicly available datasets were analyzed in this study. This data can be found here: The dataset used and analyzed in this study originate from the UK Data Service. Interested researchers can apply for access at https://beta.ukdataservice.ac.uk/datacatalogue/studies/study?id=8444#.
