# Supplementary material for: A comparative analysis of binary and multi-class classification machine learning algorithms to detect current frailty status using the English longitudinal study of ageing (ELSA)
Source: Front Aging. 2025 Apr 22;6:1501168. doi: 10.3389/fragi.2025.1501168 (PMC12052818; doi:10.3389/fragi.2025.1501168)
Supplement: Supplementary file 1 [file Table1.docx]

**Supplementary Material**

**A comparative analysis of binary and multi-class classification machine learning algorithms to detect current frailty status using the English Longitudinal Study of Ageing (ELSA)**

**Contents list**

**Supplementary Table 1.** Baseline characteristics and statistical comparisons between included and excluded participants (wave 8)

**Supplementary Table 2.** Baseline characteristics and statistical comparisons between included and excluded participants (wave 6)

**Supplementary Table 3.** Description of candidate predictors used in the binary classification models

**Supplementary Table 4.** Description of candidate predictors used in the multi-class classification models

**Supplementary Table 5.** Characterization of samples for each variable in wave 8 dataset

**Supplementary Table 6.** Characterization of samples for each variable in wave 6 dataset

**Supplementary Table 7.** Tuned hyperparameters, search space, and optimal values for tested ML models

**Supplementary Table 1.** Baseline characteristics and statistical comparisons between included and excluded participants (wave 8)

| **Variable** | **Category/Level** | **Included** | **Excluded** | **Statistical Test** | **p-value** | **Effect size** |
| --- | --- | --- | --- | --- | --- | --- |
| Age | Mean (SD) | 71.50 (7.65) | 70.90 (8.26) | -2.729 | 0.006 | -0.076 |
|  |  |  |  |  |  |  |
| Gender | Male | 2235 (44.17%) | 758 (46.93%) | 3.67 | 0.055 | 0.023 |
|  | Female | 2825 (55.83%) | 857 (53.07%) |  |  |  |
|  |  |  |  |  |  |  |
| Race | White | 4930 (97.3%) | 1550 (95.98%) | 8.64 | 0.003 | 0.036 |
|  | Non-white | 130 (2.57%) | 65 (4.02%) |  |  |  |
|  |  |  |  |  |  |  |
| Education | Less than upper secondary | 1247 (26.70%) | 494 (33.42%) | 25.00 | < 0.001 | 0.061 |
|  | Upper secondary and vocational training | 2466 (52.81%) | 707 (47.83%) |  |  |  |
|  | Tertiary | 957 (20.49%) | 277 (18.74%) |  |  |  |
|  |  |  |  |  |  |  |
| Marital status | Married/partnered | 3409 (64.71%) | 1150 (71.21%) | 12.07 | 0.007 | 0.048 |
|  | Separated/divorced | 561 (11.09%) | 135 (8.36%) |  |  |  |
|  | Widowed | 849 (16.79%) | 260 (16.10%) |  |  |  |
|  | Never married | 238 (4.71%) | 70 (4.34%) |  |  |  |
|  |  |  |  |  |  |  |
| Place of birth | Inside the UK | 4666 (92.21%) | 1438 (89.04%) | 15.36 | < 0.001 | 0.048 |
|  | Outside the UK | 394 (7.79%) | 177 (10.96%) |  |  |  |
|  |  |  |  |  |  |  |

**Supplementary Table 2.** Baseline characteristics and statistical comparisons between included and excluded participants (wave 6)

| **Variable** | **Category/Level** | **Included** | **Excluded** | **Statistical Test** | **p-value** | **Effect size** |
| --- | --- | --- | --- | --- | --- | --- |
| Age | Mean (SD) | 71.80 (8.33) | 73.49 (9.42) | -5.622 | < 0.001 | -0.190 |
|  |  |  |  |  |  |  |
| Gender | Male | 822 (47.99%) | 888 (48.90%) | 0.259 | 0.611 | 0.009 |
|  | Female | 891 (52.01%) | 928 (51.10%) |  |  |  |
|  |  |  |  |  |  |  |
| Race | White | 1665 (97.20%) | 1747 (96.25%) | 2.184 | 0.139 | 0.025 |
|  | Non-white | 48 (2.80%) | 68 (3.75%) |  |  |  |
|  |  |  |  |  |  |  |
| Education | Less than upper secondary | 623 (39.76%) | 771 (47.10%) | 17.632 | < 0.001 | 0.074 |
|  | Upper secondary and vocational training | 702 (44.80%) | 639 (39.03%) |  |  |  |
|  | Tertiary | 242 (15.44%) | 227 (13.87%) |  |  |  |
|  |  |  |  |  |  |  |
| Marital status | Married/partnered | 1145 (66.88%) | 1189 (65.47%) | 17.582 | 0.001 | 0.071 |
|  | Separated/divorced | 147 (8.59%) | 104 (5.73%) |  |  |  |
|  | Widowed | 342 (19.98%) | 440 (24.23%) |  |  |  |
|  | Never married | 78 (4.56%) | 83 (4.57%) |  |  |  |
|  |  |  |  |  |  |  |
| Place of birth | Inside the UK | 1568 (91.54%) | 1632 (90.52%) | 0.995 | 0.319 | 0.017 |
|  | Outside the UK | 145 (8.46%) | 171 (9.48%) |  |  |  |
|  |  |  |  |  |  |  |

**Supplementary Table 3.** Description of candidate predictors used in the binary classification models

| **Feature Code** | **Description** |
| --- | --- |
| Demographics | |
| rwagey | Age (years) at the time of the interview |
| raeducl | Three-tier harmonized education scale |
| Health | |
| rwshlt | Self-reported general health status |
| rwshltc | Respondent’s change in self-reported health |
| rwfallnum | The number of times the respondent has fallen down in the last 2 years |
| rwadlwaa | Whether the respondent experiences difficulties with any of the following three activities of daily living: bathing, dressing, eating |
| rwadlc | Changes in functional limitations in activities of daily living from the previous to the current interview |
| rwgrossa | Sum of the following five activities the respondent experiences difficulties with: walking 100 yards, walking across a room, climbing one flight of stairs, getting in or out of bed, bathing activities |
| rwiadlza | Sum of the following instrumented activities of daily living that the respondent has some difficulty performing: using the phone, managing money, taking medications, shopping for groceries, and preparing hot meals. |
| rwlowermoba | Whether the respondent experiences difficulties with any of the following five activities: walking 100 yards, climbing several flights of stairs without resting, getting up from a chair after sitting for long periods, and crouching |
| rwuppermoba | Whether the respondent experiences difficulties with any of the following five activities: reaching arms above shoulder level, lifting or carrying weight over 10 pounds, picking up a 5p coin from the table |
| rwgrossc | Change in functional limitations in gross motor activities indices from the previous to the current interview |
| rwarthre | Whether the respondent has reported ever having arthritis |
| rwsight | Self-rated eyesight |
| rwdsight | Self-rated distance eyesight |
| rwnsight | Self-rated near eyesight |
| rwpainlv | Usual level of pain |
| rwltactx_e | Frequency of light physical activity |
| Cognition | |
| rwtr20 | Summary score for total word recall (immediate and delayed word recall scores) |
| Income and Consumption | |
| rwissdi | Individual earnings from disability pensions (expressed as an annual equivalent in nominal pounds) |
| rwisret | Respondent’s public old-age pension (expressed as an annual equivalent in nominal pounds) |
| Retirement and Expectations | |
| rwliv10 | Self-reported probability of living to 75 (if aged under 65), 80 (if aged 66-69), 85 (if aged 70-74), 100 (if aged 85-99, 105 (if aged 100-104), 110 (if aged 105-109), or 120 (if aged 110-119) |
| rwinher | Self-reported probability of receiving an inheritance during the next 10 years |
| rwretage | Respondent’s retirement age |
| rwpnhm5y | Respondent’s probability of moving to a nursing home in the next 5 years. |
| Family Structure | |
| rwsocyr | Whether the respondent is a member of an organization, club, or society and attends at least one committee member meeting in a year |
| Assistance and Caregiving | |
| rwgkcare1w | Provided informal care to grandchildren last week |
| Stress | |
| rwksupport6 | Respondent’s lack of child support score (6-item) |
| rwosupport6 | Respondent’s lack of other family members support score (6-item) |
| rwfsupport6 | Respondent’s lack of friends support score (6-item) |
| Psychosocial | |
| rwlsatsc3 | 3-item Satisfaction with Life scale |
| rwcantril | Respondent’s rating of their place in society (10 step ladder) |
| rwcasp19 | 19-item Quality of Life scale |
| rwcesd | 8-item Center for Epidemiologic Studies Depression Scale (CES-D) |

**Supplementary Table 4.** Description of candidate predictors used in the multi-class classification models

| **Feature Code** | **Description** |
| --- | --- |
| Demographics | |
| rwagey | Age in years at the time of the current wave’s interview |
| raeducl | Three-tier harmonized education scale |
| rarelig_e | Respondent’s reported religion |
| Health | |
| rwshlt | Self-reported general health status |
| rwshltc | Change in self-reported health status from the last self-report to the current interview |
| rwadlwaa | Whether the respondent experiences difficulties with any of the following three activities of daily living: bathing, dressing, eating |
| rwadlc | Changes in functional limitations in activities of daily living from the previous to the current interview |
| rwiadlza | Sum of the following instrumented activities of daily living that the respondent has some difficulty performing: using the phone, managing money, taking medications, shopping for groceries, and preparing hot meals. |
| rwgrossa | Sum of the following five activities the respondent experiences difficulties with: walking 100 yards, walking across a room, climbing one flight of stairs, getting in or out of bed, bathing activities |
| rwgrossc | Change in functional limitations in gross motor activities indices from the previous to the current interview |
| rwlowermoba | Whether the respondent experiences difficulties with any of the following five activities: walking 100 yards, climbing several flights of stairs without resting, getting up from a chair after sitting for long periods, and crouching |
| rwuppermoba | Whether the respondent experiences difficulties with any of the following five activities: reaching arms above shoulder level, lifting or carrying weight over 10 pounds, picking up a 5p coin from the table |
| rwfallnum | The number of times the respondent has fallen down in the last 2 years |
| rwarthre | Whether the respondent has reported ever having arthritis |
| rwsight | Self-rated vision |
| rwdsight | Self-rated distance vision |
| rwnsight | Self-rated near vision |
| rwcatracte | Whether the respondent ever had cataracts |
| rwhearing | Self-rated hearing |
| rwurinai | Whether the respondent has any urinary incontinence |
| rwasthmae | Whether the respondent ever had asthma |
| rwhchole | Whether the respondent ever had high cholesterol |
| rwhibpe | Whether the respondent ever had high blood pressure |
| rwwakent_e | How often the respondent wakes up several times in the night |
| rwwakeup_e | How often the respondent has trouble staying asleep, including waking far too early |
| rwpainlv | Usual level of pain |
| rwltactx_e | Frequency of light physical activity |
| rwpsyche | Whether the respondent ever had psychological problems |
| rwsmokev | Whether the respondent has ever smoked |
| rwsmokef | Number of cigarettes the respondent smokes on average per day |
| Cognition | |
| rwtr20 | Summary score for total word recall (immediate and delayed word recall scores) |
| rworient | Summary score for orientation in time |
| Income and Consumption | |
| rwitearn | Employment earnings (after tax) |
| rwissdi | Individual earnings from disability pensions (expressed as an annual equivalent in nominal pounds) |
| rwisret | Individual earnings from public pensions without disability (expressed as an annual equivalent in nominal pounds) |
| Employment History | |
| rwlbrf_e | Respondent’s labor force status |
| rwwork | Whether the respondent is working for pay |
| Retirement and Expectations | |
| rwretage | Retirement age |
| rwpnhm5y | Self-reported probability of moving to a nursing home in the next 5 years |
| rwretrs | Main reason for retiring |
| rwearlyret | Whether the respondent took early retirement |
| rwliv10 | Self-reported probability of living to 75 (if aged under 65), 80 (if aged 66-69), 85 (if aged 70-74), 100 (if aged 85-99, 105 (if aged 100-104), 110 (if aged 105-109), or 120 (if aged 110-119) |
| Pension | |
| rwpeninm | How many occupational pensions the respondent is currently receiving income from |
| Family Structure | |
| rwkcnt | Whether the respondent has any weekly contact with children in person |
| rwcntpm | Whether the respondent has any weekly contact with relatives by phone, mail, or e-mail |
| rwfcnt | Whether the respondent has any weekly contact with friend in person |
| rwfcntpm | Whether the respondent has any weekly contact with friend by phone, mail, or e-mail |
| rwsocyr | Whether the respondent is a member of an organization, club, or society and attends at least one committee member meeting in a year |
| End of Life Planning | |
| rwlifein | Whether the respondent is covered by life insurance |
| Assistance and Caregiving | |
| rwgkcare1w | Provided informal care to grandchildren in the past week |
| Stress | |
| rwksupport6 | 6-item lack of other family members support score |
| rwosupport6 | 6-item lack of children support score |
| rwfsupport6 | 6-item lack of friend support score |
| Psychosocial | |
| rwlsatsc3 | 3-item Satisfaction with Life scale |
| rwcantril | Rating of one’s place in society (10 step ladder) |
| rwcasp19 | 19-item Quality of Life scale |
| rwcesd | 8-item Centre for Epidemiologic Studies Depression Scale |

**Supplementary Table 5.** Characterization of samples for each variable in wave 8 dataset.

| **Feature** | | **Frail**  **Mean (SD)** | **Pre-frail**  **Mean (SD)** | **Non-frail**  **Mean (SD)** | **Kruskal Statistic** | **p-value** | **Significant Dunn’s Test** | |
| --- | --- | --- | --- | --- | --- | --- | --- | --- |
| Demographics | | | | | | | | |
| r8agey | | 77.32 (8.78) | 69.35 (6.42) | 72.94 (7.82) | 443.88 | *< 0.001* | Frail vs Pre-frail  Frail vs Non-frail  Pre-frail vs Non-frail |  |
| raeducl | | 1.54 (0.62) | 2.08 (0.66) | 1.84 (0.68) | 268.271 | *< 0.001* | Frail vs Pre-frail  Frail vs Non-frail  Pre-frail vs Non-frail |  |
| rarelig_e | | 1.92 (2.31) | 2.47 (2.83) | 2.14 (2.53) | 21.971 | *< 0.001* | Frail vs Pre-frail  Pre-frail vs Non-frail |  |
| Health | | | | | | | | |
| r8shlt | | 4.03 (0.94) | 2.41 (0.92) | 3.10 (1.03) | 986.135 | *< 0.001* | Frail vs Pre-frail  Frail vs Non-frail  Pre-frail vs Non-frail |  |
| r8shltc | | 0.52 (0.72) | 0.33 (0.55) | 0.44 (0.64) | 34.741 | < 0.001 | Frail vs Pre-frail  Pre-frail vs Non-frail |  |
| r8adlwaa | | 0.55 (0.50) | 0.05 (0.22) | 0.21 (0.41) | 751.58 | *< 0.001* | Frail vs Pre-frail  Frail vs Non-frail  Pre-frail vs Non-frail |  |
| r8adlc | | 0.76 (1.12) | 0.05 (0.27) | 0.21 (0.57) | 524.949 | *< 0.001* | Frail vs Pre-frail  Frail vs Non-frail  Pre-frail vs Non-frail |  |
| r8iadlza | | 0.99 (1.27) | 0.02 (0.18) | 0.19 (0.58) | 990.824 | < 0.001 | Frail vs Pre-frail  Frail vs Non-frail  Pre-frail vs Non-frail |  |
| r8grossa | | 2.03 (1.63) | 0.09 (0.40) | 0.53 (1.01) | 1306.86 | *< 0.001* | Frail vs Pre-frail  Frail vs Non-frail  Pre-frail vs Non-frail |  |
| r8grossc | | 0.90 (1.11) | 0.06 (0.29) | 0.25 (0.62) | 655.281 | *< 0.001* | Frail vs Pre-frail  Frail vs Non-frail  Pre-frail vs Non-frail |  |
| r8lowermoba | | 2.19 (1.35) | 0.26 (0.57) | 0.79 (1.05) | 1072.187 | *< 0.001* | Frail vs Pre-frail  Frail vs Non-frail  Pre-frail vs Non-frail |  |
| r8uppermoba | | 0.74 (0.44) | 0.13 (0.34) | 0.36 (0.48) | 808.05 | *< 0.001* | Frail vs Pre-frail  Frail vs Non-frail  Pre-frail vs Non-frail |  |
| r8fallnum | | 2.68 (10.00) | 0.47 (3.14) | 0.91 (9.23) | 204.002 | < 0.001 | Frail vs Pre-frail  Frail vs Non-frail  Pre-frail vs Non-frail |  |
| r8arthre | | 0.71 (0.45) | 0.36 (0.48) | 0.53 (0.50) | 262.243 | *< 0.001* | Frail vs Pre-frail  Frail vs Non-frail  Pre-frail vs Non-frail |  |
| r8sight | | 3.18 (1.08) | 2.33 (0.87) | 2.63 (0.97) | 284.083 | *< 0.001* | Frail vs Pre-frail  Frail vs Non-frail  Pre-frail vs Non-frail |  |
| r8dsight | | 2.95 (1.11) | 2.11 (0.85) | 2.39 (0.95) | 267.892 | *< 0.001* | Frail vs Pre-frail  Frail vs Non-frail  Pre-frail vs Non-frail |  |
| r8nsight | | 3.07 (1.10) | 2.23 (0.88) | 2.50 (0.95) | 265.905 | *< 0.001* | Frail vs Pre-frail  Frail vs Non-frail  Pre-frail vs Non-frail |  |
| r8catracte | | 0.56 (0.50) | 0.30 (0.46) | 0.42 (0.49) | 141.546 | *< 0.001* | Frail vs Pre-frail  Frail vs Non-frail  Pre-frail vs Non-frail |  |
| r8hearing | | 3.10 (1.11) | 2.58 (1.02) | 2.81 (1.09) | 105.422 | *< 0.001* | Frail vs Pre-frail  Frail vs Non-frail  Pre-frail vs Non-frail |  |
| r8urinai | | 0.46 (0.50) | 0.19 (0.39) | 0.30 (0.46) | 146.658 | *< 0.001* | Frail vs Pre-frail  Frail vs Non-frail  Pre-frail vs Non-frail |  |
| r8asthmae | | 0.19 (0.39) | 0.13 (0.33) | 0.17 (0.37) | 23.506 | *< 0.001* | Frail vs Pre-frail  Pre-frail vs Non-frail |  |
| r8hchole | | 0.48 (0.50) | 0.43 (0.50) | 0.49 (0.50) | 17.490 | *< 0.001* | Pre-frail vs Non-frail |  |
| r8hibpe | | 0.63 (0.48) | 0.41 (0.49) | 0.52 (0.50) | 110.955 | *< 0.001* | Frail vs Pre-frail  Frail vs Non-frail  Pre-frail vs Non-frail |  |
| r8wakent_e | | 3.21 (1.16) | 2.93 (1.21) | 3.08 (1.18) | 34.659 | *< 0.001* | Frail vs Pre-frail  Pre-frail vs Non-frail |  |
| r8wakeup_e | | 3.09 (2.16) | 2.41 (1.74) | 2.71 (1.93) | 36.475 | *< 0.001* | Frail vs Pre-frail  Frail vs Non-frail  Pre-frail vs Non-frail |  |
| r8painlv | | 1.63 (1.17) | 0.53 (0.86) | 0.94 (1.09) | 427.506 | *< 0.001* | Frail vs Pre-frail  Frail vs Non-frail  Pre-frail vs Non-frail |  |
| r8ltactx_e | | 3.36 (1.37) | 2.17 (0.55) | 2.46 (0.95) | 557.295 | < 0.001 | Frail vs Pre-frail  Frail vs Non-frail  Pre-frail vs Non-frail |  |
| r8psyche | | 0.15 (0.36) | 0.09 (0.29) | 0.14 (0.35) | 27.758 | < 0.001 | Frail vs Pre-frail  Frail vs Non-frail  Pre-frail vs Non-frail |  |
| r8smokev | | 0.71 (0.46) | 0.61 (0.49) | 0.64 (0.48) | 14.836 | < 0.001 | Frail vs Pre-frail  Frail vs Non-frail |  |
| r8smokef | | 1.36 (5.41) | 0.52 (2.99) | 0.74 (3.53) | 20.106 | < 0.001 | Frail vs Pre-frail  Frail vs Non-frail |  |
| Cognition | | | | | | | | |
| r8tr20 | | 7.86 (3.96) | 11.58 (3.36) | 10.05 (3.54) | 435.702 | < 0.001 | Frail vs Pre-frail  Frail vs Non-frail  Pre-frail vs Non-frail |  |
| r8orient | | 3.58 (0.87) | 3.81 (0.48) | 3.77 (0.54) | 28.925 | *< 0.001* | Frail vs Pre-frail  Frail vs Non-frail  Pre-frail vs Non-frail |  |
| Income and Consumption | | | | | | | | |
| r8itearn | | 261.47 (211.78) | 2783.33 (8397.50) | 1534.13 (5423.73) | 98.136 | *< 0.001* | Frail vs Pre-frail  Frail vs Non-frail  Pre-frail vs Non-frail |  |
| r8issdi | | 1726.15 (2635.67) | 112.22 (738.70) | 511.94 (1593.88) | 564.745 | *< 0.001* | Frail vs Pre-frail  Frail vs Non-frail  Pre-frail vs Non-frail |  |
| r8isret | | 6382.01 (3370.67) | 5531.34 (3775.88) | 6091.91 (3574.51) | 30.108 | *< 0.001* | Frail vs Pre-frail  Pre-frail vs Non-frail |  |
| Employment History | | | | | | | |  |
| r8lbrf_e | | 5.03 (0.79) | 4.27 (1.63) | 4.64 (1.35) | 159.438 | *< 0.001* | Frail vs Pre-frail  Frail vs Non-frail  Pre-frail vs Non-frail |  |
| r8work | | 0.03 (0.18) | 0.25 (0.43) | 0.15 (0.36) | 155.618 | *< 0.001* | Frail vs Pre-frail  Frail vs Non-frail  Pre-frail vs Non-frail |  |
| Retirement and Expectations | | | | | | | | |
| r8retage | | 57.99 (8.73) | 60.03 (5.95) | 59.57 (7.27) | 12.813 | *< 0.001* | Frail vs Pre-frail  Frail vs Non-frail |  |
| r8pnhm5y | | 17.18 (24.87) | 9.64 (14.82) | 12.03 (17.98) | 9.240 | *< 0.001* | Frail vs Pre-frail |  |
| r8retrs | | 2.61 (1.46) | 2.88 (1.71) | 2.75 (1.60) | 10.734 | *0.004* | N/A |  |
| r8earlyret | | 0.36 (0.48) | 0.45 (0.50) | 0.43 (0.50) | 11.092 | *0.004* | N/A |  |
| r8liv10 | | 40.10 (30.62) | 63.53 (24.46) | 51.98 (27.82) | 337.557 | *< 0.001* | Frail vs Pre-frail  Frail vs Non-frail  Pre-frail vs Non-frail |  |
| Pension | | | | | | | | |
| r8peninm | | 0.86 (0.92) | 1.12 (0.98) | 1.03 (0.93) | 32.748 | *< 0.001* | Frail vs Pre-frail  Frail vs Non-frail  Pre-frail vs Non-frail |  |
| Family Structure | | | | | | | | |
| r8kcnt | | 0.85 (0.36) | 0.90 (0.30) | 0.90 (0.30) | 7.828 | *0.020* | N/A |  |
| r8rcnt | | 0.48 (0.79) | 0.50 (0.85) | 0.49 (0.84) | 13.281 | *0.001* | Frail vs Pre-frail |  |
| r8fcnt | | 0.71 (0.45) | 0.73 (0.45) | 0.72 (0.45) | 0.279 | 0.870 | N/A |  |
| r8fcntpm | | 0.61 (0.49) | 0.64 (0.48) | 0.63 (0.48) | 1.057 | 0.589 | N/A |  |
| r8socyr | | 0.18 (0.38) | 0.39 (0.49) | 0.34 (0.47) | 57.357 | *< 0.001* | Frail vs Pre-frail  Frail vs Non-frail  Pre-frail vs Non-frail |  |
| End of Life Planning | | | | | | | |  |
| r8lifein | | 0.25 (0.43) | 0.23 (0.42) | 0.23 (0.42) | 1.056 | 0.590 | N/A |  |
| Assistance and Caregiving | | | | | | | | |
| r8gkcare1w | | 0.02 (0.15) | 0.06 (0.23) | 0.05 (0.22) | 7.682 | 0.021 | N/A |  |
| Stress | | | | | | | | |
| r8ksupport6 | | 1.80 (0.66) | 1.61 (0.49) | 1.69 (0.54) | 31.975 | *< 0.001* | Frail vs Pre-frail  Pre-frail vs Non-frail |  |
| r8osupport6 | | 1.96 (0.67) | 1.89 (0.61) | 1.94 (0.64) | 6.035 | *0.049* | N/A |  |
| r8fsupport6 | | 1.73 (0.53) | 1.63 (0.44) | 1.69 (0.48) | 16.924 | *< 0.001* | Frail vs Pre-frail  Pre-frail vs Non-frail |  |
| Psychosocial | | | | | | | | |
| r8lsatsc3 | | 2.25 (0.67) | 2.73 (0.47) | 2.52 (0.61) | 279.379 | *< 0.001* | Frail vs Pre-frail  Frail vs Non-frail  Pre-frail vs Non-frail | |
| r8cantril | | 5.31 (1.67) | 6.41 (1.50) | 5.88 (1.65) | 195.895 | *< 0.001* | Frail vs Pre-frail  Frail vs Non-frail  Pre-frail vs Non-frail | |
| r8casp19 | | 32.80 (7.85) | 45.05 (7.07) | 39.70 (8.66) | 723.103 | *< 0.001* | Frail vs Pre-frail  Frail vs Non-frail  Pre-frail vs Non-frail | |
| r8cesd | | 3.26 (2.09) | 0.52 (0.86) | 1.79 (1.95) | 1207.609 | *< 0.001* | Frail vs Pre-frail  Frail vs Non-frail | |
|  |  |  |  |  |  |  |  | |

**Supplementary Table 6.** Characterization of samples for each variable in wave 6 dataset

| **Feature** | | **Frail**  **Mean (SD)** | **Pre-frail**  **Mean (SD)** | **Non-frail**  **Mean (SD)** | **Kruskal Statistic** | **p-value** | **Significant Dunn’s Test** | |
| --- | --- | --- | --- | --- | --- | --- | --- | --- |
| Demographics | | | | | | | | |
| r6agey | | 79.56 (8.51) | 69.66 (7.24) | 74.13 (8.74) | 312.813 | *< 0.001* | Frail vs Pre-frail  Frail vs Non-frail  Pre-frail vs Non-frail |  |
| raeducl | | 1.42 (0.62) | 1.91 (0.72) | 1.61 (0.66) | 141.281 | *< 0.001* | Frail vs Pre-frail  Frail vs Non-frail  Pre-frail vs Non-frail |  |
| rarelig_e | | 1.77 (2.10) | 2.25 (2.65) | 2.01 (2.40) | 5.391 | 0.067 | N/A |  |
| Health | | | | | | | | |
| r6shlt | | 4.09 (0.93) | 2.59 (0.95) | 3.29 (1.05) | 474.299 | *< 0.001* | Frail vs Pre-frail  Frail vs Non-frail  Pre-frail vs Non-frail |  |
| r6shltc | | 0.48 (0.69) | 0.40 (0.60) | 0.48 (0.70) | 3.054 | 0.217 | N/A |  |
| r6adlwaa | | 0.56 (0.50) | 0.07 (0.26) | 0.25 (0.43) | 350.594 | *< 0.001* | Frail vs Pre-frail  Frail vs Non-frail  Pre-frail vs Non-frail |  |
| r6adlc | | 0.84 (1.15) | 0.05 (0.27) | 0.26 (0.60) | 287.329 | *< 0.001* | Frail vs Pre-frail  Frail vs Non-frail  Pre-frail vs Non-frail |  |
| r6iadlza | | 1.20 (1.32) | 0.07 (0.42) | 0.30 (0.71) | 505.111 | < 0.001 | Frail vs Pre-frail  Frail vs Non-frail  Pre-frail vs Non-frail |  |
| r6grossa | | 2.41 (1.58) | 0.12 (0.50) | 0.76 (1.13) | 798.021 | *< 0.001* | Frail vs Pre-frail  Frail vs Non-frail  Pre-frail vs Non-frail |  |
| r6grossc | | 1.12 (1.21) | 0.07 (0.33) | 0.38 (0.72) | 412.306 | *< 0.001* | Frail vs Pre-frail  Frail vs Non-frail  Pre-frail vs Non-frail |  |
| r6lowermoba | | 2.56 (1.21) | 0.29 (0.64) | 1.09 (1.17) | 763.437 | *< 0.001* | Frail vs Pre-frail  Frail vs Non-frail  Pre-frail vs Non-frail |  |
| r6uppermoba | | 0.84 (0.37) | 0.15 (0.36) | 0.47 (0.50) | 529.171 | *< 0.001* | Frail vs Pre-frail  Frail vs Non-frail  Pre-frail vs Non-frail |  |
| r6fallnum | | 1.91 (4.23) | 0.37 (2.23) | 1.45 (13.92) | 194.419 | < 0.001 | Frail vs Pre-frail  Frail vs Non-frail  Pre-frail vs Non-frail |  |
| r6arthre | | 0.70 (0.46) | 0.32 (0.47) | 0.51 (0.50) | 152.105 | *< 0.001* | Frail vs Pre-frail  Frail vs Non-frail  Pre-frail vs Non-frail |  |
| r6sight | | 3.30 (1.11) | 2.37 (0.87) | 2.84 (1.05) | 203.211 | *< 0.001* | Frail vs Pre-frail  Frail vs Non-frail  Pre-frail vs Non-frail |  |
| r6dsight | | 3.06 (1.15) | 2.15 (0.84) | 2.60 (1.04) | 191.671 | *< 0.001* | Frail vs Pre-frail  Frail vs Non-frail  Pre-frail vs Non-frail |  |
| r6nsight | | 3.21 (1.15) | 2.30 (0.87) | 2.71 (1.02) | 183.728 | *< 0.001* | Frail vs Pre-frail  Frail vs Non-frail  Pre-frail vs Non-frail |  |
| r6catracte | | 0.59 (0.49) | 0.22 (0.42) | 0.42 (0.49) | 167.470 | *< 0.001* | Frail vs Pre-frail  Frail vs Non-frail  Pre-frail vs Non-frail |  |
| r6hearing | | 3.25 (1.13) | 2.59 (1.06) | 2.90 (1.12) | 87.002 | *< 0.001* | Frail vs Pre-frail  Frail vs Non-frail  Pre-frail vs Non-frail |  |
| r6urinai | | 0.35 (0.48) | 0.11 (0.31) | 0.19 (0.39) | 96.528 | *< 0.001* | Frail vs Pre-frail  Frail vs Non-frail  Pre-frail vs Non-frail |  |
| r6asthmae | | 0.19 (0.39) | 0.10 (0.30) | 0.17 (0.38) | 23.581 | *< 0.001* | Frail vs Pre-frail  Pre-frail vs Non-frail |  |
| r6hchole | | 0.49 (0.50) | 0.40 (0.49) | 0.41 (0.49) | 8.911 | *0.012* | Frail vs Pre-frail  Frail vs Non-frail |  |
| r6hibpe | | 0.66 (0.47) | 0.46 (0.50) | 0.52 (0.50) | 38.961 | *< 0.001* | Frail vs Pre-frail  Frail vs Non-frail  Pre-frail vs Non-frail |  |
| r6wakent_e | | 3.28 (1.15) | 2.86 (1.24) | 3.09 (1.19) | 38.583 | *< 0.001* | Frail vs Pre-frail  Frail vs Non-frail  Pre-frail vs Non-frail |  |
| r6wakeup_e | | 3.13 (2.16) | 2.20 (1.69) | 2.62 (1.94) | 44.863 | *< 0.001* | Frail vs Pre-frail  Frail vs Non-frail  Pre-frail vs Non-frail |  |
| r6painlv | | 1.52 (1.21) | 0.50 (0.85) | 1.01 (1.11) | 224.296 | *< 0.001* | Frail vs Pre-frail  Frail vs Non-frail  Pre-frail vs Non-frail |  |
| r6ltactx_e | | 3.37 (1.36) | 2.19 (0.58) | 2.67 (1.12) | 420.772 | < 0.001 | Frail vs Pre-frail  Frail vs Non-frail  Pre-frail vs Non-frail |  |
| r6psyche | | 0.11 (0.32) | 0.08 (0.27) | 0.11 (0.32) | 6.366 | 0.042 | N/A |  |
| r6smokev | | 0.71 (0.45) | 0.65 (0.48) | 0.70 (0.46) | 6.780 | 0.034 | N/A |  |
| r6smokef | | 1.60 (4.82) | 0.90 (3.77) | 1.14 (4.56) | 7.697 | 0.021 | Frail vs Pre-frail |  |
| Cognition | | | | | | | | |
| r6tr20 | | 6.47 (3.74) | 10.54 (3.40) | 8.76 (3.72) | 290.357 | < 0.001 | Frail vs Pre-frail  Frail vs Non-frail  Pre-frail vs Non-frail |  |
| r6orient | | 3.29 (1.04) | 3.81 (0.51) | 3.60 (0.73) | 118.205 | *< 0.001* | Frail vs Pre-frail  Frail vs Non-frail  Pre-frail vs Non-frail |  |
| Income and Consumption | | | | | | | | |
| r6itearn | | 76.11 (660.36) | 2397.02 (6913.24) | 1326.74 (6231.39) | 83.277 | *< 0.001* | Frail vs Pre-frail  Frail vs Non-frail  Pre-frail vs Non-frail |  |
| r6issdi | | 2079.43 (2817.29) | 166.81 (926.99) | 722.40 (1692.72) | 342.631 | *< 0.001* | Frail vs Pre-frail  Frail vs Non-frail  Pre-frail vs Non-frail |  |
| r6isret | | 6982.43 (3836.77) | 5114.47 (3515.94) | 5740.28 (3310.51) | 52.582 | *< 0.001* | Frail vs Pre-frail  Frail vs Non-frail  Pre-frail vs Non-frail |  |
| Employment History | | | | | | | |  |
| r6lbrf_e | | 5.12 (0.56) | 4.24 (1.67) | 4.78 (1.26) | 130.846 | *< 0.001* | Frail vs Pre-frail  Frail vs Non-frail  Pre-frail vs Non-frail |  |
| r6work | | 0.01 (0.10) | 0.26 (0.44) | 0.11 (0.31) | 146.919 | *< 0.001* | Frail vs Pre-frail  Frail vs Non-frail  Pre-frail vs Non-frail |  |
| Retirement and Expectations | | | | | | | | |
| r6retage | | 58.79 (8.83) | 59.62 (6.98) | 59.11 (7.29) | 2.552 | 0.279 | N/A |  |
| r6pnhm5y | | 18.68 (28.12) | 8.02 (14.79) | 12.56 (20.49) | 16.164 | *< 0.001* | Frail vs Pre-frail  Pre-frail vs Non-frail |  |
| r6retrs | | 2.68 (1.45) | 2.79 (1.66) | 2.73 (1.53) | 0.540 | 0.763 | N/A |  |
| r6earlyret | | 0.41 (0.49) | 0.50 (0.50) | 0.51 (0.50) | 8.509 | *0.001* | N/A |  |
| r6liv10 | | 34.99 (32.85) | 60.07 (26.48) | 48.05 (30.59) | 158.838 | *< 0.001* | Frail vs Pre-frail  Frail vs Non-frail  Pre-frail vs Non-frail |  |
| Pension | | | | | | | | |
| r6eninm | | 0.79 (0.81) | 0.97 (0.90) | 0.88 (0.83) | 11.586 | *0.003* | Frail vs Pre-frail |  |
| Family Structure | | | | | | | | |
| r6kcnt | | 0.90 (0.30) | 0.88 (0.33) | 0.90 (0.30) | 1.824 | 0.402 | N/A |  |
| r6rcnt | | 0.68 (0.47) | 0.57 (0.49) | 0.60 (0.49) | 7.478 | *0.024* | Frail vs Pre-frail |  |
| r6fcnt | | 0.72 (0.45) | 0.71 (0.46) | 0.68 (0.47) | 1.825 | 0.401 | N/A |  |
| r6fcntpm | | 0.59 (0.49) | 0.60 (0.49) | 0.57 (0.50) | 1.954 | 0.376 | N/A |  |
| r6socyr | | 0.13 (0.34) | 0.36 (0.48) | 0.24 (0.43) | 50.254 | *< 0.001* | Frail vs Pre-frail  Pre-frail vs Non-frail |  |
| End of Life Planning | | | | | | | |  |
| r6lifein | | 0.30 (0.46) | 0.27 (0.45) | 0.27 (0.45) | 1.198 | 0.549 | N/A |  |
| Assistance and Caregiving | | | | | | | | |
| r6gkcare1w | | 0.01 (0.09) | 0.07 (0.25) | 0.04 (0.20) | 14.725 | *< 0.001* | N/A |  |
| Stress | | | | | | | | |
| r6ksupport6 | | 1.68 (0.57) | 1.59 (0.50) | 1.64 (0.50) | 4.129 | 4.129 | N/A |  |
| r6osupport6 | | 1.90 (0.61) | 1.88 (0.63) | 1.87 (0.61) | 0.415 | 0.813 | N/A |  |
| r6fsupport6 | | 1.73 (0.51) | 1.68 (0.46) | 1.71 (0.51) | 1.457 | 0.483 | N/A |  |
| Psychosocial | | | | | | | | |
| r6lsatsc3 | | 2.08 (0.70) | 2.67 (0.51) | 2.40 (0.67) | 169.625 | *< 0.001* | Frail vs Pre-frail  Frail vs Non-frail  Pre-frail vs Non-frail | |
| r6cantril | | 5.24 (1.83) | 6.01 (1.60) | 5.49 (1.77) | 45.913 | *< 0.001* | Frail vs Pre-frail  Pre-frail vs Non-frail | |
| r6casp19 | | 30.56 (8.26) | 43.36 (7.18) | 36.87 (8.63) | 389.741 | *< 0.001* | Frail vs Pre-frail  Frail vs Non-frail  Pre-frail vs Non-frail | |
| r6cesd | | 3.48 (2.15) | 0.47 (0.83) | 1.93 (2.05) | 696.246 | *< 0.001* | Frail vs Pre-frail  Frail vs Non-frail  Pre-frail vs Non-frail | |
|  |  |  |  |  |  |  |  | |

**Supplementary Table 7.** Tuned hyperparameters, search space, and optimal values for tested ML models

|  | **Hyperparameter** | **Search Space** | **Multi-class Models:**  **Selected value** | **Binary Models: Selected value** |
| --- | --- | --- | --- | --- |
|  |  |  |  |  |
| Logistic Regression | C | 0.001, 0.01, 0.1, 1, 10, 100 | 0.1 | 1 |
|  | penalty | l1, l2 | l1 | l2 |
|  | solver | liblinear, saga | saga | saga |
| Random Forest | n_estimators | 50, 100, 200, 300 | 300 | 200 |
|  | max_depth | none, 10, 20, 30, 40 | none | none |
|  | min_samples_split | 2, 5, 10 | 2 | 2 |
|  | min_samples_leaf | 1, 2, 4 | 1 | 1 |
|  | bootstrap | true, false | false | false |
| K-nearest Neighbor | n_neighbors | 3, 5, 7, 10 | 7 | 3 |
|  | weights | uniform, distance | distance | uniform |
|  | p | 1, 2 | 1 | 1 |
| Gradient Boosting | n_estimators | 50, 100, 200 | 200 | 200 |
|  | learning_rate | 0.001, 0.01, 0.1, 0.2 | 0.2 | 0.2 |
|  | max_depth | 3, 5, 7 | 7 | 3 |
|  | subsample | 0.8, 0.9, 1.0 | 0.9 | 0.8 |
| AdaBoost | n_estimators | 50, 100, 200 | 200 | 200 |
|  | learning_rate | 0.01, 0.1, 0.5 | 0.5 | 0.5 |
|  | max_depth | SAMME, SAMME.R | SAMME | SAMME |
| XGBoost | n_estimators | 50, 100, 200 | 200 | 200 |
|  | learning_rate | 0.01, 0.1, 0.2 | 0.2 | 0.2 |
|  | max_depth | 3, 5, 7 | 7 | 3 |
|  | subsample | 0.8, 0.9, 1.0 | 0.9 | 0.9 |
|  | colsample_bytree | 0.8, 0.9, 1.0 | 1.0 | 1.0 |
|  | gamma | 0, 0.1, 0.2 | 0 | 0 |
| LightGBM | n_estimators | 50, 100, 200 | 200 | 200 |
|  | learning_rate | 0.001, 0.01, 0.1, 0.2 | 0.2 | 0.1 |
|  | max_depth | -1, 10, 20, 30 | -1 | -1 |
|  | num_leaves | 31, 63, 127 | 127 | 31 |
|  | boosting_type | gbdt, dart, goss | gbdt | gbdt |
| CatBoost | iterations | 50, 100, 200 | 200 | 200 |
|  | learning_rate | 0.01, 0.1, 0.2 | 0.2 | 0.1 |
|  | depth | 6, 8, 10 | 10 | 6 |
|  | l2_leaf_reg | 1, 3, 5 | 1 | 1 |
| Multi-layer Perceptron | hidden_layer_sizes | (50,), (100,), (50, 50), (100, 50) | (50, 50) | (50,) |
|  | activation | tanh, relu, logistic | Logistic | Relu |
|  | solver | sgd, adam | Sgd | Adam |
|  | alpha | 0.01, 0.001, 0.0001 | 0.0001 | 0.01 |
|  | learning_rate | constant, adaptive | constant | constant |
|  |  |  |  |  |
